# Research on the resilience of renewable energy in rural areas—Based on the data from Shandong Province

**DOI:** 10.1371/journal.pone.0329097

**Published:** 2025-08-06

**Authors:** Zihan Ma

**Affiliations:** School of Economics and Management, North China Electric Power University, Baoding, China; Lanzhou Jiaotong University, CHINA

## Abstract

China’s rural energy system faces three challenges: high structural carbonization, low energy utilization efficiency and insufficient supply stability. These systemic contradictions seriously hinder the coordinated promotion of the “dual carbon” strategy and the rural revitalization process. This study takes 16 cities and cities in Shandong Province as the object and combines GIS technology to construct a biomass and solar energy resource evaluation model. The amount of agricultural biomass resources is calculated through the grass valley ratio method. Its resource potential and convertible power generation are quantified, and the “Four Quadrant Model for Renewable Energy Abundance” is introduced to divide regional types. Use spatial analysis to reveal the geographical heterogeneity of resource distribution and explore differentiated low-carbon transformation paths to enhance energy resilience. The study found that the rural renewable energy endowment in Shandong Province showed significant regional differences and proposed four types of development paths: The dual-resource areas jointly develop agricultural and light complementarity and straw power generation, the photovoltaic advantage zone explores energy storage and hydrogen production, the biomass-led areas strengthen cogeneration, and the resource-scarce areas implement green electricity allocation and energy efficiency upgrades. The conclusion shows that multi-energy coordination can improve energy supply stability through space-time complementarity and risk dilution, enhance energy supply resilience, and provide a scientific paradigm for the low-carbon transformation of high-carbon provinces.

## 1. Introduction

As a major agricultural country, my country’s rural areas occupy an important position in the national energy system, and their energy structure and utilization efficiency issues need to be solved urgently. First, the rural population base and energy consumption scale are large. Taking the data from 2022 as an example, the rural population is as high as 490 million, accounting for 34.78% of the total population in the country [1]. Although the total rural energy consumption accounts for only 15% of the country, it still has 230 million tons of standard coal equivalent after conversion [2]. The huge scale of energy consumption has aggravated the negative impacts of problems such as high carbonization and low utilization efficiency in rural areas and restricted rural development. Second, in the rural energy structure, the contradiction between high carbonization and inefficiency in utilization is extremely prominent. Rural scattered coal consumption and direct combustion utilization methods still account for a high proportion. This “high consumption-low efficiency-strong emission” model not only leads to inefficient energy utilization but also causes serious air pollution, which has had many adverse effects on the health of villagers. Third, the energy infrastructure in rural areas is not sound and the pressure on energy transformation is relatively high. This backward energy utilization model runs contrary to my country’s philosophy of pursuing green and sustainable development and seriously hinders the pace of rural areas to move towards modernization [3]. The existence of these problems has made the optimization of rural energy structure and the improvement of utilization efficiency an important issue that needs to be solved urgently.

Under the guidance of the “dual carbon” goal, enhancing the supply capacity of rural renewable energy and promoting low-carbon development in rural areas has become a top priority. First, my country’s rural areas have rich renewable energy resources and great development and utilization potential. Agricultural waste such as straw, rice husks, fruit husks, etc., and livestock and poultry breeding manure are huge and can be used as raw materials for biomass power generation and biogas production. Rural areas have vast land and low building density, providing unique conditions for the development of solar and wind energy. If these biomass resources can be effectively converted, they can not only become clean energy but also reduce environmental pollution [4]. Second, the country attaches importance to the development of rural renewable energy. In recent years, the government has introduced a series of policies to support the development of rural clean energy. In 2022, the State Council clearly stated in the “Opinions on Doing a Good Job in Comprehensively Promoting Rural Revitalization Key Work”, that it is necessary to vigorously promote the construction of clean energy such as photovoltaics and biomass energy in rural areas [5]. This policy orientation has pointed out the direction for the development of rural clean energy. In addition, the “14th Five-Year Plan for Modern Energy System” also redefined rural areas as clean energy supply sites in the modern energy system [6]. With the support of policies, various regions have increased their investment in rural clean energy projects. Many rural areas have begun to build photovoltaic power stations on a large scale. The government has provided certain subsidies and preferential policies to encourage villagers to participate in photovoltaic power generation projects, which not only increases villagers’ income but also promotes the popularization of clean energy in rural areas [7].Third, the development of rural renewable energy is of great significance to the realization of rural revitalization and the realization of the “dual carbon” goals. With the optimization of rural energy structure, the widespread application of renewable energy will promote the upgrading of rural industrial structure and promote the diversified development of the rural economy [8].In addition, the development and utilization of renewable energy can improve the self-sufficiency rate of rural energy, enhance the resilience of the energy supply, reduce dependence on traditional fossil energy, and further reduce carbon emissions [9]. This green transformation not only helps achieve sustainable development in rural areas but also provides important support for the achievement of the country’s “dual carbon” goals.

As a major energy-consuming province in my country, Shandong Province plays an important role in energy consumption and carbon emissions in its rural areas, but it also faces many challenges. First, energy consumption and carbon emissions account for a high proportion. In 2023, Shandong Province’s energy consumption reached 526 million tons of standard coal equivalent [10], and both energy consumption and carbon emissions account for about 10% of the country [11]. Among them, the proportion of total energy consumption and carbon emissions in rural areas is high, and structural contradictions are prominent. Traditional fossil energy still dominates the rural energy structure. The excessive dependence on traditional fossil energy not only leads to inefficient energy utilization but also makes carbon emissions remain high. A large amount of coal combustion produces pollutants such as CO_2_ and SO_2_, which have caused serious damage to the air quality and ecological environment in rural areas. Second, renewable energy development potential is great but the utilization rate is low. As a major agricultural province, Shandong Province has great potential for renewable energy. Taking biomass energy as an example, 70 million tons of crop straw are generated annually [10], and the theoretical biomass energy potential reaches 35 million tons of standard coal equivalent, but the actual energy conversion rate is less than 30%. This means that a large amount of biomass resources have been wasted and failed to be effectively utilized. In addition, the rural roof photovoltaic development area in the province exceeds 280 million m^2^ [10], with a theoretical installed capacity of 56 GW, but the current development rate is only 12.5%. Third, the problem of energy time and space mismatch is prominent. Traditional energy systems show obvious vulnerability in the periodic fluctuations of “summers are abundant and winters are dry, the day is full and nights are scarce” [12]. In summer, electricity demand in rural areas is relatively low, while in winter, heating demand increases greatly, and electricity supply is often not enough to meet the demand; during the day, the photovoltaic power generation is relatively large, but at this time, the villagers’ electricity demand is relatively low, and the electricity demand in the night increases, but photovoltaic power generation cannot be met. This mismatch not only affects residents’ quality of life to a certain extent, but also limits the development of some industries in rural areas that rely on energy supply, hinders the pace of rural areas’ transformation towards green and low-carbon transformation, and is not conducive to the implementation of local sustainable development strategies.

In recent years, rural energy issues have attracted widespread attention from many scholars at home and abroad, and in-depth research has been carried out from different angles. Han (2021) conducted in-depth research on factors affecting energy consumption. By constructing a complex economic model, collecting a large amount of energy consumption data and economic development indicator data, and conducting correlation analysis and regression analysis. His research results point out that carbon emissions are in line with the environmental Kuznets curve assumption, that is, as the level of economic development increases, carbon emissions will first rise and then fall [13]. Wang (2017) selected 120 villages for surveys. By designing a detailed questionnaire, he went deep into rural families communicated with villagers face to face, and analyzed the current status of rural household energy consumption and its influencing factors in detail. He found that factors such as household income level, population structure, and energy prices have a significant impact on energy consumption [14]. Tian Yun et al. (2023) used the autocorrelation model and STIRPAT extension model to conduct spatial autocorrelation analysis and decomposition of influencing factors on a large number of rural carbon emission data, revealing the spatial and temporal pattern of rural carbon emissions and found that there is obvious spatial agglomeration phenomenon in rural carbon emissions in different regions, and the impact of factors such as economic development, population size, and technical level on carbon emissions vary in different periods [15]. Zhang et al. (2014) accurately estimate rural energy carbon emissions from the perspective of the life cycle, from the entire life cycle of energy extraction, transportation, and processing to final use, considering carbon emissions in each link, providing a new perspective for a comprehensive understanding of rural energy carbon emission [16]. Mainali et al. (2014) have introduced energy sustainability indexes to build a multi-dimensional indicator system covering energy supply stability, energy utilization efficiency, environmental impact, etc., to evaluate the sustainability of rural household energy in developing countries, and to show the current development status and progress of various countries in this regard through principal component analysis, providing a quantitative basis for developing countries to formulate rural energy development policies [17]. Wang Ping et al. (2023) went deep into rural areas of Shaanxi and Henan to conduct household surveys, taking zero carbonization of living energy as the starting point, and analyzing the current situation of daily energy consumption such as cooking, heating, lighting, etc. in rural households, they explored the path to achieve the “carbon peak” of living energy, and proposed measures such as promoting clean energy equipment and strengthening energy management [18]. Jiang Yi et al. (2022) discussed the application potential of a new rural energy system based on rural roof photovoltaic systems and its important role in clean energy consumption and environmental protection. Through actual case analysis and technical and economic evaluation, they pointed out that rural roof photovoltaic systems have huge potential to reduce rural energy carbon emissions and improve rural environmental quality [19]. Shen Ruixia et al. (2022) conducted research on the issue of daily energy consumption in rural Heilongjiang. By collecting historical energy consumption data and using time series analysis and other methods, they predicted and analyzed the changing trends in their consumption and energy structure, providing a reference for Heilongjiang’s rural energy planning [20]. Xiao Rui et al. (2023) used the super-efficiency SBM model to measure the efficiency of rural energy carbon emissions in my country and analyzed the nonlinear impact relationship between residents’ income level and rural energy carbon emissions efficiency through the panel Tobit model. They found that residents’ income level has a positive impact on rural energy carbon emissions efficiency within a certain range, but the impact weakens after exceeding a certain threshold [21]. Liu Zhixiong (2019) took the microdata of households in rural areas of Beijing, Tianjin, and Hebei as a sample, and explored the factors that affect the consumption of traditional biomass energy in rural households from the perspective of consumers. By constructing a consumer behavior model, he found that factors such as energy prices, traditional habits, and environmental awareness have an important impact on traditional biomass energy consumption [22].Wang Qiang et al. (2022) study pointed out that biomass coal substitutes for coal” can effectively reduce carbon emissions, encourage consumers to participate in “carbon neutrality”, and promote rural energy transformation [23].

In recent years, domestic and foreign scholars and organizations have gradually paid attention to the construction of a sustainable renewable energy system and the improvement of energy resilience. Gatto and Drago (2020) argue that the ability of energy systems to withstand, respond to, overcome and transcend damage caused by shocks in multiple fields (e.g., environmental, social, economic and institutional) is known as energy resilience [24].In its 2020 Sustainable Recovery report, IEA mentioned a global energy sector sustainable recovery plan that has three goals, including increasing the resilience and sustainability of energy systems. Plans to reduce the risk of power outages, increase flexibility, reduce losses, and help integrate more variable renewable energy sources to increase the resilience of the energy system and enable the energy system to withstand future shocks by investing in better grids and increasing efficiency [25].Osman et al. (2023), biomass can be efficiently converted into energy forms such as electricity, biodiesel and biohydrogen through thermochemistry and bioconversion technologies. As the only renewable energy form with carbon neutrality, its integration in power production can not only alleviate the problem of energy shortage, but also significantly reduce greenhouse gas emissions through the carbon cycle mechanism. Its technical path and integration model have become the research focus of academic and industrial circles [26].In the 2023 Agricultural Energy Policy Guidelines, FAO clearly states that energy resilience includes infrastructure disaster resilience such as grid wind disaster resistance design and socio-economic adaptability (such as farmer energy diversification strategies). The ESF program is coordinated with the Hand-in-Hand Initiative to optimize energy-agricultural investment through geospatial data analysis. This helps to some extent improve the ability of agricultural energy systems to meet various challenges, reflecting the concern about energy resilience [27].Ahmed et al. (2024) studies the storage systems of renewable energy from the perspective of energy management to balance the production and consumption storage of various renewable energy sources. Provides insights into all aspects of community energy for sustainable energy transition, and provides an in-depth review of energy communities, especially renewable energy communities, highlighting the importance of civic participation [28].Zhang et al. (2024) focuses on the application of renewable energy systems in a certain industry, especially the latest developments in renewable energy systems used for building heating, cooling and thermal energy storage power generation [29].Osman et al. (2022) further focused on the possibility that climate change may affect the stability of renewable energy systems through extreme weather and long-term environmental pressures. Rural decentralized energy infrastructure is more sensitive to climate disturbance than centralized systems [30].Shafiei et al. (2024) systematically integrates renewable energy (RES) and battery energy storage systems (BESS) into the power grid, focusing on elastic metrics that involve multi-objective optimization methods that consider the relative battery capacity and total system cost [31].

Comprehensive consideration of the above studies has found that the above studies have certain limitations. Specifically, although the above research has paid attention to the supply of renewable energy in rural areas and its influencing factors, and has made progress in evaluating energy resilience, they are lacking in some key aspects. First, Domestic research mostly ignores the support role of local coordinated utilization of renewable energy on the stability of the entire energy supply structure. Second, the existing international framework is mostly based on the experience of developed countries, and lacks guidance on the differentiated path design of rural energy systems in high-carbon provinces.When formulating development paths, we failed to adapt to local conditions well; Third, its focus is mainly focused on the national or provincial level, and there is a lack of sufficient consideration for more micro and targeted situations. Fourth, there is little research on energy resilience in rural areas and the impact of climate change has not been fully considered in policy implementation.

In order to overcome the shortcomings of the above research,this study proposes GIS and the ‘four-quadrant classification model’ in method. Through spatial heterogeneity analysis and multi-energy complementarity mechanism, it makes up for the gap between the international framework in the construction of a renewable energy system. This paper takes Shandong Province, China as a research case, evaluates the amount of rural renewable energy resources from the perspective of renewable energy elasticity, and considers the stability of the energy system under the influence of climate.,and explores a development path to maximize the synergistic effect of renewable energy, so as to cope with the risk of energy gap caused by insufficient energy allocation, improve energy resilience, and provide key support for the deep integration of rural revitalization and the dual-carbon strategy,and provide a replicable resilience improvement paradigm for similar regions around the world.

## 2. Methodology

### 2.1 Research regions

This article selects Shandong Province as a research case and refines the research to 16 prefecture-level cities in Shandong Province. The main reasons are as follows: First, it is representative of research. Shandong is not only a traditional energy province but also has rich renewable energy resources. Shandong Province has abundant renewable energy resources. Light resources rank among the top in the country and are suitable for the large-scale development of photovoltaics; as a major agricultural province, the annual output of crop straw biomass energy reaches 70 million tons [10], providing a foundation for the research on the multi-energy complementary model of renewable energy. At the same time, Shandong Province was once the largest coal consumption province in the country, with a high proportion of rural scattered coal heating, significant pressure on carbon emissions, high dependence on traditional energy, and urgent demand for transformation [32]. Studying its path to transition to renewable energy can provide a “low-carbon breakthrough” sample for high-carbon provinces. Second, it is very feasible. Shandong Province is the first country’s county-wide photovoltaic pilot and biomass energy clean heating demonstration province. It has formed a practical foundation with a new energy and renewable energy power generation installed capacity of 115GW and a photovoltaic installed capacity ranked first in the country [33] and has observable and quantifiable research conditions. Third, it has scientific research. Shandong’s GDP ranks third in the country, but urban and rural development is unbalanced and has great regional differences. From coastal to inland, the economic and technical levels are different. Such diversity is suitable for scientific analysis of energy resilience, the research and design are more scientific, and the results are more convincing. Therefore, this research field is representative, feasible, and scientific.

### 2.2 Methods

**Fig 1:**
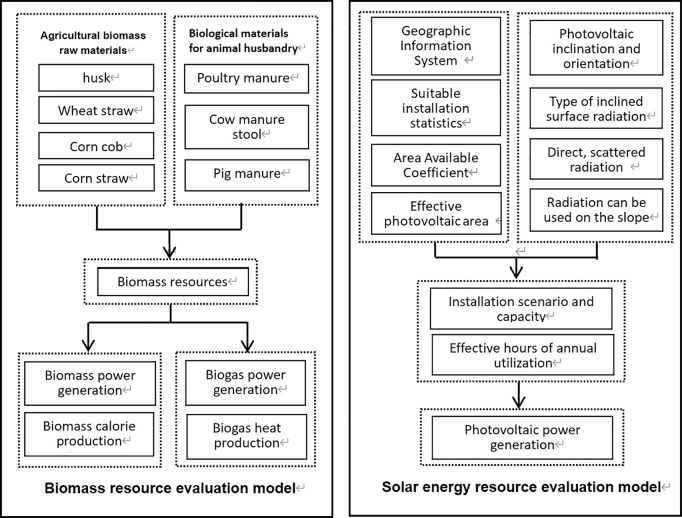
Renewable energy assessment model in rural areas of Shandong Province.

This section is based on geographic information system (GIS) technology and builds a multi-dimensional resource evaluation model for the two most widely distributed renewable energy sources in rural areas. This model realizes quantitative calculations of resource physical reserves, thermal energy potential, and power generation potential through three major modules: spatial data fusion, resource density inversion, and energy conversion simulation. The specific technical framework is shown in Fig 1.

#### 2.2.1 Agricultural biomass resource evaluation model.

When exploring the utilization of agricultural biomass resources in rural areas in depth, the model constructed considers the fields of agriculture and animal husbandry, involving a total of 7 kinds of biomass raw materials. These key data selections are derived from the “Shandong Province Statistical Yearbook (2024)” [10]. When estimating the available resources for biomass raw materials, the production data of agriculture and animal husbandry published in the “Shandong Provincial Statistical Yearbook” covers key indicators such as crop yield, as well as livestock and output of livestock. On this basis, we use the calculation method of grass-grain ratio, biomass resource coefficient, and net calorific value to learn from relevant domestic and foreign literature [34–37] to ensure the accuracy and reliability of the data. The technical potential of biomass raw materials for direct combustion and heat generation of industrial boilers, direct combustion power generation, direct combustion heat generation of biogas boilers, and biogas power generation is evaluated based on the resource volume and the conversion efficiency of different technologies [38–40].

The formula for the production of heat of biomass is as follows:


$$BEC_{ij}=RP_{j}\times NCV_{j}\times \frac{1}{1000}$$ (1)

Where: i represents different cities, with a total of 16; j represents biomass energy varieties, with a total of 7 categories; it is the j-th biomass energy available in the city of i in the year, GJ; RP_j_ is the resource potential of j-th biomass energy, t; NCV_j_ is the net calorific value of j-th biomass energy, MJ·kg^-1^.

The thermal energy potential generated by biogas combustion is evaluated according to the following formula:


$$BGTE_{ij}=RP_{j}\times \;\%VS_{j}\times BGP_{j}\times D\times EC\times \frac{1}{10^{6}}$$ (2)

Where: BGTE_ij_ is the potential of the biomass energy conversion biogas thermal energy in i city, GJ; RP_j_ is the resource potential of the jth biomass energy, t; %VSj is the proportion of volatile solids of the j-th biomass, %; BGP_j_ is the ratio of methane to volatile solids produced by j-th biomass, m^3^/kg; D is the biogas concentration, with a value of 1.15 kg·m^-3^; EC is the energy density of biogas, with a value of 50.4 TJ·kg^-1^.

The biomass power generation potential is evaluated according to the following formula:


$$E_{b}=BGTE_{ij}\times \eta_{1}\times \eta_{2}\times \eta_{3}$$ (3)

Where: E_b_ is the amount of biomass power generation (unit is kwh), η_1_ is the efficiency of converting biomass energy into thermal energy, η_2_ is the efficiency of converting heat energy into mechanical energy, and η_3_ is the efficiency of converting mechanical energy into electrical energy. According to relevant research literature [3] and conservative estimates [36], η_1_ × η_2_ × η_3_ = η takes a value of 25%.

The biogas power generation formula is as follows:


$$E_{bg}=BGTE_{ij}\times \eta_{4}\times \eta_{5}$$ (4)

Where: E_bg_ is the amount of biogas power generation (units kwh), η_4_ is the efficiency of converting heat energy into mechanical energy in biogas combustion, and η_5_ is the efficiency of converting mechanical energy into electrical energy. According to relevant research literature [38–40] and conservative estimates, η_4_ × η_5_ = η takes a value of 35%.

The parameters required for the model are shown in Table 1 and Table 2.

**Table 1:** Correlation coefficient of agricultural biomass assessment.

Remaining raw materials	Biomass resource coefficient	Collectable utilization rate(%)	Net calorific value(MJ·kg^-1^)
Crouch	0.27	95	14
Wheat straw	1.2	76	15.363
Corn core	0.25	97	14.395
Corn straw	1.2	95	15.539

**Table 2:** Correlation coefficient of biomass assessment in animal husbandry.

Remaining raw materials	Collect coefficients	Volatile solids	Ratio of methane to volatile solid (m^3^/kg)	Biogas concentration (kg·m^-3^)	Biogas energy density (TJ· kg^-1^)	Breeding cycle (days)	Exhaust coefficient
Pig manure	0.9	0.6	0.42	1.15	50.4	199	1.5
Cow manure stool	0.6	0.6	0.3	1.15	50.4	365	23.4
Poultry manure	0.6	0.6	0.49	1.15	50.4	210	0.1

#### 2.2.2 Solar energy resource evaluation model.

This study’s assessment of the technological potential of solar energy resources focuses on the field of photovoltaic power generation. Through a three-dimensional framework of geospatial analysis, radiation data modeling, and system efficiency correction, a refined evaluation model is constructed. The specific methods are as follows:

Use ArcGIS (version 10.8) software [41] and map data files to remove areas that are not suitable for photovoltaic installation, such as forests, cultivated land, and land with a slope of more than 15%, from the total area of Shandong Province, cut the remaining areas into each city, and calculate the area suitable for the development and utilization of solar energy in ArcGIS.

According to the Global Solar Atlas [41,42], the amount of solar radiation irradiated to a flat surface at an optimal inclination angle is obtained, i.e., Global Inclined Radiation (GTI), in units of kWh/m^2^/y. The potential of photovoltaic power generation is developed based on a simulation model of the universal crystalline silicon (c-Si) module, which considers local conditions such as losses caused by temperature or pollution, and trims the Rizhao raster dataset to the boundary of Shandong Province to calculate the average value of each city.

The potential of photovoltaic power generation is obtained from the available area of photovoltaic, annual inclined radiation, and comprehensive efficiency coefficient estimates. The calculation formula is:


$$E_{pv}=S\times GTI\times K\times 10^{-6}$$ (5)

Where: E_pv_ is the photovoltaic power generation potential, GWh; S is the available area, m^2^; GTI is the annual inclined radiation value, kwh/m^2^/y; K is the comprehensive efficiency coefficient, with a value of 79.1% [43].

#### 2.2.3 Energy resilience indicator development.

This study is based on the theory of system dynamics and risk management, and regards the energy system as a dynamic adaptation system, covering the entire cycle of “immunity-maintenance-recovery”. The design of redundancy index draws on the theory of system redundancy and points out the ability of quantitative energy structures to diversify risks through multi-energy complementarity. The specific development indicators are shown in Table 3.

**Table 3:** Energy resilience indicators.

Indicator dimensions	Quantitative method	Source of data
**reliability**	Renewable energy annual power generation volatility (standard deviation/mean)	Photovoltaic and biomass power generation potential data in each city
**Redundancy**	Multi-energy complementary index = (1 – single energy share) * 100%	Energy combination types in each region
**Adaptability**	Energy supply retention rate under extreme climate events (historical power outages/renewable energy system recovery time)	Extreme weather data of Shandong Provincial Meteorological Bureau
**recovery time**	Time required to restore to 90% of normal energy supply after system interruption (hours)	Grid fault repair records, biomass raw material supply chain response speed

## 3. Results

### 3.1 Evaluation of agricultural biomass resources

The systematic evaluation of biomass resources in rural areas of Shandong Province in 2023 is shown in Table 4. The total biomass resources in the province reached 71.49 million tons, showing significant agricultural resources. Among them, agricultural biomass is 62.88 million tons, accounting for as much as 88%, which is the core pillar of renewable energy development. Further analysis shows that straw resources occupy an absolutely dominant position, with a total volume of 55.28 million tons, accounting for 87.9% of the total agricultural biomass, mainly including 24.38 million tons of wheat straw and 30.8948 million tons of corn straw. The total biomass in the animal husbandry industry is 8.6077 million tons, the biogas heat production is 1440636.42 TJ, and the biogas power generation potential is 140061 GWh. Mainly it is 5.05 million tons of poultry manure and 2.75 million tons of cow manure, its highly volatile solid content provides high-quality raw materials for biogas development.

**Table 4:** Shandong Province Biomass Resource Evaluation.

Material raw materials		Resources (10,000t)	Biomass calorie production (TJ)	Biomass power generation (GWH)	Biogas heat production (TJ)	Biogas power generation (GWH)
agriculture	Crouch	102.83	14396.84	999.78	–	–
	Wheat straw	2438.47	374622.47	26015.45	–	–
	Corn core	657.19	94602.78	6569.64	–	–
	Corn straw	3089.48	480074.23	33338.49	–	–
Animal Husbandry	Pig manure	80.17	–	–	292756.01	28462.39
	Cow manure stool	275.42	–	–	287336.53	27935.50
	Poultry manure	505.01	–	–	860543.88	83663.99
Total		7148.57	963696.33	66923.36	1440636.42	140061.87

**Fig 2:**
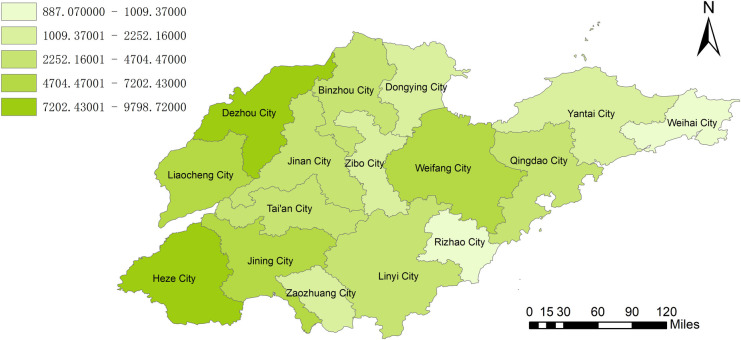
Power generation potential of agricultural biomass resources in Shandong Province.

**Fig 3:**
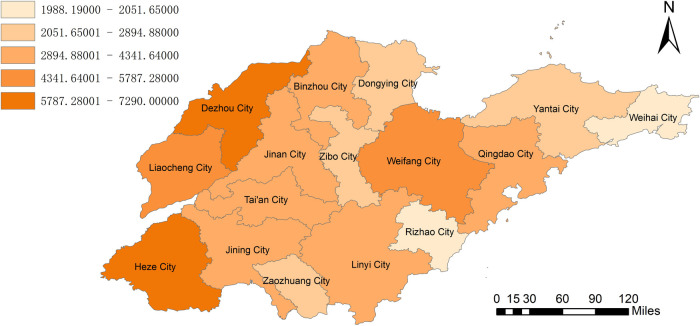
Power generation potential of biomass resources in animal husbandry in Shandong Province.

**Fig 4:**
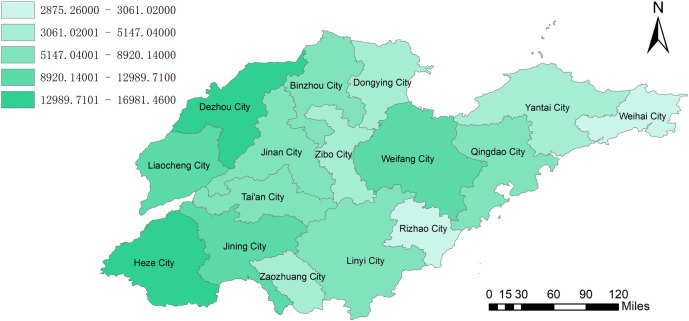
Total power generation potential of biomass resources in Shandong Province.

By combining the geographic information system to analyze the amount of biomass resources, we can clearly observe from Fig 2 that Dezhou City and Heze City have unique advantages in agricultural biomass resources. With its vast arable land area, diversified crop planting, and advanced agricultural production system, Dezhou City has accumulated extremely rich agricultural biomass resources, and its power generation potential is as high as 9659 GWH. Heze City also has extremely abundant agricultural biomass resources, with a power generation potential of 9799 GWH. Fig 3 clearly shows the geographical distribution of animal husbandry biomass resources. It can be clearly seen that Dezhou City and Heze City also dominate in this field. The livestock industry’s potential for power generation in Dezhou can reach 7290 GWh. Heze City’s livestock industry has a potential of 7183 GWh. According to the geographical distribution of the total power generation potential of biomass resources in Fig 4, it was found that Heze City has the highest total power generation potential in the province, reaching 16982GWH, and it has made concerted efforts in the development and utilization of biomass resources in agriculture and animal husbandry. Weihai City and Rizhao City seem to be relatively poor in terms of biomass resources. Weihai City’s power generation potential is only 2875 GWh. Rizhao City also has only 3061GWH in power generation potential. In the future energy development plan, these two cities need to fully consider their own resource shortcomings and actively explore diversified energy development paths to make up for the energy supply gap caused by the scarcity of biomass resources.

### 3.2 Solar energy resource evaluation

**Fig 5:**
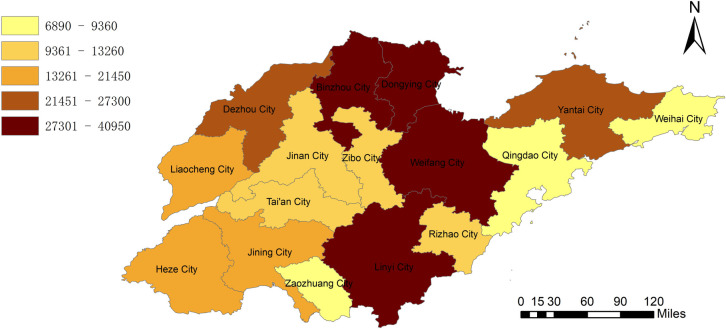
Potential of solar energy resources in Shandong Province.

Based on the evaluation results in Fig 5, it can be seen that Shandong Province has distinct regional characteristics in the field of solar energy development. Across the province, areas suitable for solar energy development are mainly concentrated in the central and western regions. It is estimated that the area of this area is about 13,970 km^2^, and the vast land provides unique conditions for the large-scale development of solar energy, demonstrating its huge advantages in the construction of solar power generation hardware facilities. In terms of annual power generation potential, it has performed excellently, reaching 272,610GWh, which means that the region can contribute considerable electricity to the province’s energy supply every year with solar power generation. The distribution of solar energy resources in Shandong Province is presented in Fig 5. First, among the cities in the province, Dongying City and the central and northern regions around Weifang City stand out, and its solar energy utilization potential is at the top. In this area, sufficient lighting time, a good geographical environment, and suitable climatic conditions have jointly created its ultra-high solar energy development value. After an in-depth evaluation, the region can generate up to 40,950GWh of electricity per year, becoming the core area and pillar force of solar power generation in Shandong Province. Secondly, Weihai City and Zaozhuang City are at a low level in the province in terms of solar energy resources. Due to the combined influence of various factors such as its geographical location, topography, and climate, Weihai City has relatively little solar radiation, resulting in limited solar power generation potential, and its annual power generation potential is only 6890 GWh. Zaozhuang City also faces a similar dilemma. Due to its own natural conditions, the availability of solar energy resources is not high, and the annual power generation potential is only 8190 GWh. Compared with the surrounding areas of Dongying and Weifang in solar energy development, the gap between these two cities and cities is relatively obvious. In the future energy development plan, it is necessary to explore more suitable energy development paths based on their own actual conditions to make up for the energy supply shortcomings caused by insufficient solar energy resources.

### 3.3 Analysis of renewable energy abundance

**Fig 6:**
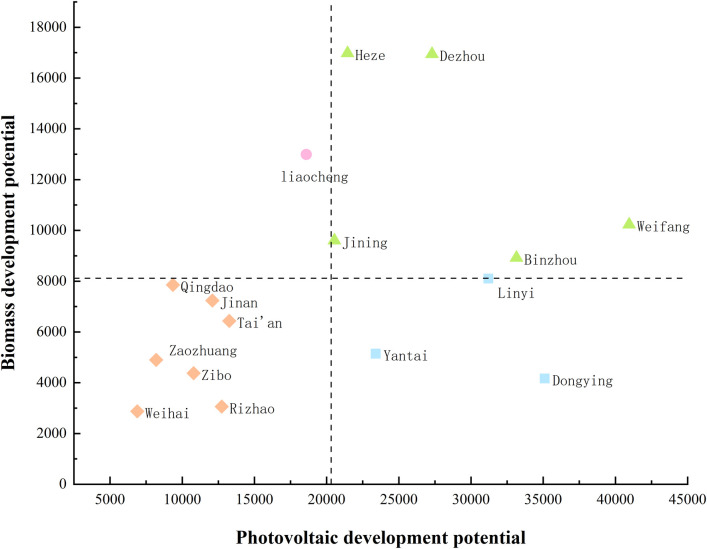
Schematic diagram of the development potential of biomass and photovoltaic resources in rural areas of Shandong cities.

**Fig 7:**
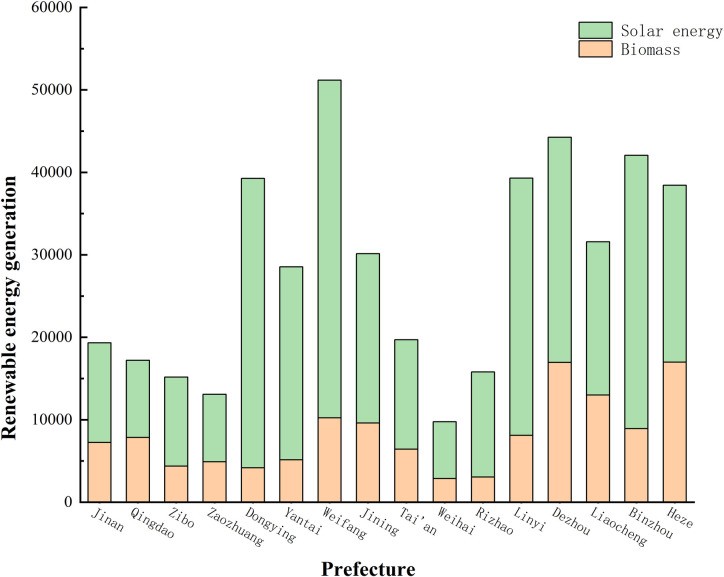
The potential of agricultural biomass and photovoltaic resource power generation in rural areas of various cities in Shandong Province.

Based on the potential for development and utilization and the power generation capacity used as a key measurement indicator, the renewable energy resources in 16 cities and cities in Shandong Province are visually accumulated, as shown in Fig 7. The results clearly show the unique advantages and development potential of cities in the fields of renewable energy such as solar energy and biomass energy. In order to more intuitively present the renewable energy abundance in various cities in Shandong Province, we take solar energy development potential as the horizontal axis and biomass development potential as the vertical axis, as shown in Fig 6. In this chart, the distribution of renewable energy resources in each city is clear at a glance, providing an important visual basis for further analysis and decision-making. Fig 7 focuses on the power generation potential of biomass and solar energy resources in rural areas of various cities and cities. With its unique solar energy resources, Weifang City occupies first place in the field of renewable energy power generation, with renewable energy power generation of up to 51182 GWh, becoming the leader in the province. Dezhou City followed closely behind, with renewable energy generation reaching 44249 GWh. Weihai City, Zaozhuang City, and other cities have relatively weak endowments in solar energy and biomass resources, and their renewable energy power generation is at a low level, which is relatively low in the province’s ranking.

In order to analyze the renewable energy utilization potential of 16 cities and cities in a more in-depth manner, we innovatively divide the two variables, solar energy, and biomass energy, into four distinct sectors.

The specific division basis is as follows:

1. Use the improved mean method for solar power generation threshold, and add ± 10% buffer zone based on the average of solar power generation in 16 cities to avoid “one size fits all”
2. Biomass: Median-quantile mixing method

Threshold: 8000 GHz is set when close to the mean but avoiding the influence of right bias

① First calculate the upper quartile (Q3 = 9604 GWh) and divide the biomass potential into:

High potential: ≥ Q3 (Jining, Weifang, Dezhou, Heze, Liaocheng)

Medium potential: Q1~Q3 (Q1=4901 GWh)

Low Potential: < Q1

② Additional requirements for high-potential zones: ≥ Provincial planning target value (8500 GWh)

Through the above threshold division, biomass uses quantile law to avoid the impact of right-bias distribution, and achieves anti-outlier interference space-policy coordination: setting buffer zones and exception rules to improve classification inclusion is a system of energy elastic management.

[1] Double-high potential sector. This sector, composed of Weifang City, Jining City, Binzhou City, Heze City, and Dezhou City, has shown extremely high development potential in both biomass resources and solar energy resources. In the field of biomass power generation, these cities and cities have a high power generation potential of up to 62687 GWh with their abundant biomass raw materials such as crop straw, livestock manure, and advanced biomass power generation technology. In terms of solar power generation, the vast land resources and sufficient lighting conditions make its solar power generation potential reach 143390 GWh. Adding up the two, the total annual power generation of this sector is as high as 206077 GWh, which is undoubtedly the core area of renewable energy development in Shandong Province and has superior conditions for large-scale development of biomass and solar power generation.
[2] Advantage sectors of solar energy. Yantai City, Linyi City, and Dongying City have relatively few biomass resources, but they have unique advantages in solar energy resources. Among them, Dongying City is the region with the richest solar energy resources. With the full use of existing technologies and resources, its solar power generation potential can reach 35100 GWh. The biomass potential for the entire sector is 17420 GWh, while the solar power generation potential is as high as 89700 GWh, with an annual power generation potential of 107120 GWh. In the future, this sector can focus on the development of the solar power generation industry, and further enhance the proportion and benefits of solar power generation through technological innovation and scale expansion.
[3] Biomass Advantage Section. This section composed of Liaocheng City shows the significant characteristics of abundant biomass resources and relatively scarce solar energy resources. Liaocheng’s developed characteristic agriculture provides rich raw materials for the development of biomass energy, such as various specialty agricultural product processing waste, special energy crops, etc. After these resources are converted, the power generation potential can reach 12990 GWh. Although solar energy resources are limited, by rationally laying out solar power facilities, 18590 GWh of electricity can be provided to the local area every year, and a total of 31580 GWh of electricity can be generated in total. For Liaocheng City, we should focus on deepening the field of biomass energy power generation, and at the same time moderately develop solar energy resources to achieve efficient utilization and sustainable development of energy.
[4] Relatively scarce sectors. The sector consists of Jinan City, Qingdao City, Zibo City, Zaozhuang City, Tai’an City, Weihai City, and Rizhao City. The urbanization rate in most areas is relatively high, and the number of rural residents is relatively small, which also leads to the relative scarcity of biomass and solar energy resources. According to statistics, the biomass power generation potential of the seven prefectures and cities in this sector accounts for only 28% of the 16 prefectures and cities in the province, and the solar power generation potential accounts for only 22%. Although resources are relatively scarce, these cities and cities can rely on their advanced scientific and technological level and strong economic strength to improve energy utilization efficiency through technological innovation and energy management optimization and explore renewable energy development paths suitable for themselves, such as developing distributed energy systems and promoting the application of energy storage technology.

### 3.4 Renewable energy resilience analysis

Through the analysis of Table 5 and Table 6, the data in each dimension point to “multi-energy complementarity + localized resources + operation and maintenance capabilities” are the core elements to improve energy resilience, and there is significant correlation between the data, such as the regional adaptability loss rate with high redundancy is lower and the recovery time is shorter, which further strengthens the argument that photovoltaic and biomass multi-energy complementarity improves energy resilience. At the same time, this energy resilience index system has some limitations, such as not covering the impact of energy storage systems on resilience; extreme climate scenario simulation only involves sandstorms and heavy rainstorms, and lacks scene data such as ice and snow, high temperatures, etc., which can be used as a direction for future research.

**Table 5:** Quantitative analysis of rural energy resilience in Shandong Province.

Indicator dimensions	Specific indicators	Double high potential zone	Photovoltaic advantage zone	Biomass Advantage Zone	Resource-scarce areas	Source of data
**Reliability analysis**	Photovoltaic power generation summer and winter volatility	35%	58%	–	–	Monthly photovoltaic power generation data of 16 prefectures in Shandong Province (Provincial Energy Bureau 2023 Monitoring Report)
	Change rate of power generation in winter and summer of biomass energy	+28%	–	+28%	–	Statistics of the quarterly biomass energy output in Shandong Province (2024 data from the Provincial Department of Agriculture and Rural Affairs)
	Total regional energy volatility	18.2%	42.7%	14.5%	–	Heze/ Dongying/ Liaocheng Energy Supply and Demand Monitoring Data (2023 Report of Municipal Development and Reform Commission)
**Redundancy Assessment Multi-energy**	Complementary Index	82%	55%	70%	31%	Shandong Province’s Energy Structure Statistics (Provincial Statistics Bureau’s “2024 Shandong Energy Balance Table”)
	Biomass redundancy (Northwest Shandong)	0.85	–	0.85	–	Dezhou/ Liaocheng straw resource density measurement (2024 Journal of Shandong Agricultural Sciences)
	Distributed photovoltaic coverage (Jiaodong Peninsula)	–	35%	–	12%	Qingdao/ Weihai Rural Photovoltaic Installation Statistics (2023 registered data from Municipal Energy Bureau)
**Adaptation verification**	Dust Storm Photovoltaic Efficiency Decrease Rate (Dezhou)	–	23%	–	–	Dezhou Photovoltaic Power Station Operation Log (2023 Extreme Weather Report)
	Biomass power generation efficiency decline rate in rainy season (Heze)	18%	–	18%	–	Heze Biomass Power Station Fault Record (Operation and Maintenance Report from June to August 2023)
	Energy supply loss rate for a single disaster	≤20%	45%−60%	30%−40%	≥70%	Simulation results of Shandong Province’s energy resilience assessment model (Provincial Development and Reform Commission 2024 Technical Report)
**Recovery time simulation**	Photovoltaic panel damage repair cycle	7-15 days	7-15 days	–	7-15 days	Shandong Photovoltaic Operation and Maintenance Enterprise Service Data (Surveyed by Provincial Renewable Energy Association in 2023)
	Biomass supply chain recovery time	72 hours	–	72 hours	–	Liaocheng/Dezhou Distributed Storage Station Scheduling Record (2023 Emergency Drill Report)
	Regional comprehensive recovery time	48 hours	96 hours	60 hours	120 hours	Jining/Zaozhuang Energy Emergency Response Assessment (2024 Plan Acceptance Data of Municipal Emergency Management Bureau)

**Table 6:** Quantitative summary of rural energy resilience in Shandong Province.

Indicator dimensions	Double-high potential zone (Heze, Weifang, Jining)	Single/ scarce zone (Dongying, Weihai, Zaozhuang)
**reliability**	Volatility: 18.2% (synergy reduction: 49%)	Volatility in the photovoltaic area is 42.7%, and the power generation in winter has dropped sharply to 28%.
**Redundancy**	Multi-energy complementarity index 82%, straw density ≥ 3t/ha	External dependence 69%, PV coverage rate 12%
**Adaptability**	Disaster loss rate is ≤ 20%, and the multi-energy buffering effect is significant	Loss rate ≥45%, weak risk resistance of a single energy
**recovery time**	48 hours recovery (localized operation and maintenance + multi-energy switching)	120 hours of recovery (relying on external support + technology shortage)

## 4. Discussion

This study constructed a renewable energy assessment model, combined with the GIS system, and evaluated biomass and solar energy resources in rural areas of 16 prefectures and cities in Shandong Province. By analyzing and studying the abundance of renewable energy in rural areas of each city, we found that due to factors such as urbanization rate and geographical location, the distribution of renewable energy resources in rural areas of Shandong Province has obvious geographical differences. Through the analysis of the four sectors of renewable energy abundance, it can be seen that rural areas such as Weifang City, Jining City, Binzhou City, Heze City, Dezhou City, and other cities have advantages in both biomass and photovoltaic resources. Yantai City, Linyi City, Dongying City, and other cities have relatively few biomass resources, but they have rich photovoltaic resources. Liaocheng City is rich in biomass resources, while photovoltaic resources are relatively scarce. In contrast, the biomass resources and photovoltaic resources in Jinan City, Qingdao City, Zibo City, Zaozhuang City, Tai’an City, Weihai City, and Rizhao City are not abundant.

Making full use of renewable energy is an important way to achieve low-carbon development in rural areas. Strengthening green and low-carbon development in rural areas of Shandong Province must work together from multiple parties. First of all, the energy supply side should be developed in individual regions. Weifang, Jining, Binzhou, Heze, Dezhou, and other cities have both excellent biomass and solar energy resources, with an annual power generation potential of more than 200,000 GWh. It can coordinate the development of agricultural and optical complementary power stations and straw power generation, forming a three-dimensional energy supply network of “field energy + roof photovoltaic”; Yantai, Linyi, and Dongying solar energy leading areas focus on photovoltaics and coastal wind power hydrogen production in Dongying saline-alkali land, and explore the “photo-hydrogen storage integration” model; Liaocheng relies on characteristic agriculture to develop biomass cogeneration, and moderately supplement distributed photovoltaic; Due to limited resources and high urbanization rate, seven cities including Jinan and Qingdao need to strengthen cross-regional green power allocation and energy efficiency improvement. By introducing green power in western Shandong and northern Shandong and promoting building photovoltaics, they will reduce their dependence on traditional energy. Secondly, it is necessary to promote green and low-carbon development in rural areas on the energy consumption side and coordinate the coordinated transformation of multiple fields such as construction, transportation, and agricultural production. In terms of architecture, photovoltaic technology can be combined with energy-saving transformation to promote the self-sufficiency of renewable energy through integrated building photovoltaic design and reduce traditional energy dependence with measures such as optimization of enclosure structures and popularization of clean cookware. The transportation field should focus on guiding electrification replacement, gradually eliminating high-emission vehicles, and exploring the application of clean energy such as biomass fuels in transportation scenarios. Agricultural production needs to accelerate the promotion of electrification technology, reduce the scale of use of diesel machinery, promote the integration of new energy equipment such as photovoltaic irrigation and intelligent temperature control with agricultural facilities, and build a low-carbon production system. Through technology application, equipment update, and energy consumption model optimization, rural energy utilization efficiency will be systematically improved, and the goal of achieving the overall low-carbon transformation. In addition, the high urbanization rate of Qingdao and Jinan cities has a limited proportion of rural areas, and overall carbon emissions are becoming stable, so the results of emission reduction need to be further consolidated. It is recommended to strengthen clean energy substitution on the supply side, expand the scale of distributed photovoltaics and industrial waste heat utilization; deepen building energy-saving transformation and transportation electrification transformation on the consumption side, and strictly control the re-burning of scattered coal and the rebound of high-energy-consuming industries. Relying on the advantages of wind and light resource enrichment, such as Dezhou and Dongying, we can accelerate the construction of centralized photovoltaic bases and offshore wind power projects, and provide supporting energy storage facilities to improve the green electricity consumption capacity. Through technical support and financial incentives, we will build a rural zero-carbon energy demonstration zone and strive to achieve a regional carbon peak by 2028; it is expected to achieve 342 million tons of CO_2_e emission reduction by 2035. Resource-constrained regions such as Zaozhuang and Weihai focus on strengthening the control of energy consumption intensity and implementing two-way regulation of “scenery and light increment + traditional reduction”. Give priority to the development of distributed energy such as roof photovoltaics and biomass heating, and gradually reduce the proportion of coal in rural heating and small industries. Simultaneously promote the cross-regional green electricity trading mechanism, rely on the Jiaodong Economic Circle energy collaboration network to make up for local resource shortcomings, and ensure that it is achieved simultaneously with the province’s carbon peak target.

Technological innovation is the key to making full use of renewable energy and improving energy conversion efficiency from the root. Osman et al. conducted relevant in-depth research and provided ideas for innovation in biomass technology transformation. The biomass cogeneration and cogeneration systems can be integrated with the cogeneration system, and the biomass cogeneration (CHP) system can be promoted, and the turbine power generation can be driven by combustion or gasification of biomass can be generated, while the waste heat is recovered for heating. Cogeneration technologies can be developed, such as the integrated model of “biomass gasification-synthesis gas power generation-biocarbon carbon sequestration”, which not only improves energy utilization, but also achieves negative carbon emissions through biochar storage. Upgrade combustion and gasification technology to reduce pollutant emissions research, adopt low-nitrogen combustion technology (such as hierarchical combustion, recombustion technology) and efficient dust removal equipment (such as electrostatic dust collectors) to reduce pollutant emissions during the combustion process. Promote gasification power generation technology, such as circulating fluidized bed gasification furnaces, the synthesis gas generated can be used for power generation by gas turbines after purification, reducing greenhouse gas emissions by 25% compared to direct combustion; develop collaborative application of biochar and expand heating scenarios, improve biochar as heating fuel, perform torrefaction pretreatment of agricultural waste, produce high-density biochar fuel, and be used for heating of household stoves or industrial boilers, and replace part of coal burning.

Research is not limited to exploring the effects of a single energy supply for renewable energy. Regional-scale climate vulnerability assessment and multi-energy complementary design are the core of improving rural energy resilience. Faced with uncertainty in climatic conditions, typhoons and salt spray in coastal areas of Shandong Province (such as Weihai and Rizhao) may accelerate photovoltaic module corrosion, while sandstorm weather in inland areas (such as Dezhou and Weifang) will increase cleaning costs. This study recommends exploring the “photo-hydrogen storage integration” model in photovoltaic advantageous areas (such as Dongying), to stabilize sunshine fluctuations through energy storage systems, and to use hydrogen energy to enhance system climate adaptability. As a major agricultural province, extreme precipitation may cause mildew in straw to reduce calorie value or livestock and poultry epidemics, affecting feces supply. In this regard, the dual-high potential zone needs to establish a distributed biomass warehousing network and promote stress-resistant crop varieties such as drought-resistant corn to improve the resilience of the raw material supply chain. Therefore, improving rural energy resilience requires taking into account resource endowment and climate adaptability, and hedging the climate vulnerability of a single energy through multi-energy complementarity. Research has found that achieving excellent results in the coordinated use of multi-energy renewable energy can further improve the resilience of the energy supply. First of all, photovoltaic energy and biomass energy have significant dynamic complementarity at the space-time level. Photovoltaic energy relies on solar radiation cycles, and its intermittent characteristics form a natural coupling relationship with the continuous and stable output of biomass energy. Through inverse phase fluctuation verification (correlation coefficient), gap filling rate calculation, system reliability improvement triple-quantitative analysis, combined with empirical project data, photovoltaic and biomass energy has been strictly proven to have dynamic time complementarity in Shandong Province.This characteristic is particularly evident in rural areas of Shandong Province. After data simulation analysis, photovoltaic power generation is high in summer and low in winter, and biomass energy, inversely, forming inverse phase fluctuations of seasonal growth and growth characteristics. Biomass energy power generation increased by 28% in winter compared to 8% in summer, directly filling the photovoltaic gap.For example, when photovoltaic power generation gradually decreases due to weakening light, biomass energy can effectively fill the intermittent gap in photovoltaic power generation with its raw material storage and flexible scheduling mechanism, thus forming a time-based all-weather energy supply chain. The biomass energy output is stable, with a fluctuation of less than 15% throughout the year, which can hedge the seasonality of photovoltaics exceeding 100% violent fluctuations. The data simulation shows that the correlation between the two is −0.72, indicating that the two are highly seasonal complementary.From a spatial perspective, photovoltaic systems are adapted to areas with abundant light resources, while biomass energy can be widely distributed based on the agricultural and forestry waste resource network. Taking 16 cities and cities in Shandong Province as an example, by constructing a renewable energy assessment model and combining the GIS system to evaluate biomass and solar energy resources in rural areas of each city, it was found that due to factors such as urbanization rate and geographical location, the distribution of renewable energy resources in rural areas of Shandong Province has obvious geographical differences. Rural areas of Weifang City, Jining City, Binzhou City, Heze City, Dezhou City, and other cities have advantages in biomass and photovoltaic resources, which enables these areas to better achieve spatial coordination between photovoltaic energy and biomass energy, optimize the spatial allocation efficiency of regional energy, and form a three-dimensional energy supply network of “field energy + roof photovoltaics”, with annual power generation potential exceeding 200,000 GWh. Secondly, in the face of disturbing scenarios such as extreme climates or equipment failures, the dual energy module can achieve risk dilution through dynamic load allocation. This advantage is fully reflected in the energy supply practice in rural areas of Shandong Province. When photovoltaic arrays are partially damaged in disasters, biomass energy can maintain the basic load through distributed energy supply nodes to ensure the basic energy supply. On the contrary, when the biomass raw material supply chain is blocked for some reason, photovoltaic power generation can quickly respond to demand peaks. This two-way fault tolerance mechanism significantly reduces the risk of systemic collapse caused by single-point failure and provides solid guarantees for a stable energy supply in rural areas. Third, the collaborative model of biomass energy and photovoltaic energy innovatively integrates energy production and ecological circulation. Follow the principle of circular economy to achieve efficient utilization and closed-loop flow of resources, promote decarbonization of the energy system through the zero-carbon regional framework, and the two jointly support the sustainability goal of “reducing external resource dependence”. First of all, at the resource level, reduce the input of fossil energy and improve the waste resource utilization rate. Secondly, the environmental level: reduce carbon emissions and help regional carbon neutrality; the third economic level: form a synergistic effect of the industrial chain and create green employment and economic value.In rural areas of Shandong Province, this integration has brought many positive effects. On the one hand, energy crops can be planted in the space under the photovoltaic panels, which not only improves the compound utilization rate of the land but also provides rich raw materials for the biomass system. Achieve industrial coordination and build a circular economy industrial chain. The solar energy and biomass energy industry can form a cross-industry cycle: the photovoltaic industry provides clean energy for biomass processing (such as power supply of biogas fermentation equipment); the slag and wood ash generated by the biomass energy industry can be used as agricultural fertilizers to feed back to the farmland around the photovoltaic panels, forming a circular ecology of “photovoltaic + agriculture + energy”.On the other hand, the thermal energy margin generated during biomass conversion can reverse the optimization of the operating temperature environment of the photovoltaic module. For example, Liaocheng City has abundant biomass resources. By developing biomass cogeneration and moderately supplementing the cloth photovoltaics, in the energy production process, the thermal energy generated by the conversion of biomass energy can be used to regulate the temperature around the photovoltaic module and improve the power generation efficiency of the photovoltaic module. The energy crops planted under the photovoltaic panels provide raw materials for biomass energy production, forming a good material-energy dual cycle. Both solar energy and biomass energy are renewable resources, and their comprehensive use can help replace fossil energy sources, such as coal and natural gas, thereby reducing dependence on external energy. The circular economy emphasizes “input-end reduction”, and the large-scale application of renewable energy directly reduces external links such as energy extraction and transportation, and reduces the system’s demand for natural resources. This design makes the system’s dependence on external resource inputs continue to decrease and promotes the sustainable development of energy in rural areas.

According to the research, we provide personalized development suggestions for the resilience of energy supply in 16 cities in Shandong Province to expand the research results to the application level and promote the formation of a new energy governance paradigm through the energy synergy model. The positive impact of this energy synergy model goes far beyond the energy sector. From a social perspective, it has effectively spawned a social network of green employment and the diffusion of low-carbon technologies. In the biomass energy conversion and the construction and operation and maintenance of distributed photovoltaic facilities, a large number of jobs have been created, covering multiple fields from technology research and development, equipment manufacturing, project installation to daily operation and maintenance, providing local residents with rich employment opportunities, especially attracting some migrant workers to return to their hometowns to find jobs, and promoting economic development and social stability in rural areas. At the same time, with the widespread application and promotion of these green energy technologies, relevant low-carbon technology knowledge has been rapidly disseminated in the community, which has stimulated residents’ awareness and interest in green energy and further promoted the popularization of low-carbon lifestyles in the whole society. From the perspective of multi-dimensional technology-economic-society, this energy collaboration model has successfully built a solid resilience base, providing valuable practical examples and development ideas for the sustainable development of energy and green transformation of the social economy in Shandong Province and even the whole country.

However, the construction of renewable energy systems faces multiple constraints. Based on the research situation and policy recommendations given, we discuss possible model restrictions and policy implementation obstacles in order to be universal and scientific.

First of all, at the institutional level, rural renewable energy projects often face cross-departmental coordination problems. Taking Shandong Province’s biomass power generation project as an example, straw collection and storage involve supervision from the Agriculture and Rural Affairs Bureau. Power generation and grid connection need to be coordinated with the Energy Bureau and the power grid enterprises, and environmental emissions are under the jurisdiction of the ecological environment department. This management pattern can easily lead to a long approval process conflict with regulatory standards. In addition, the county-level policy implementation capabilities vary significantly. Agricultural counties in northwestern Shandong have a dependence on central fiscal subsidies as high as 73%, affecting the sustainability of the project.

Secondly, the public’s acceptance of renewable energy and energy system changes also affects the energy transformation process, the construction of renewable energy systems, and the ministry of various cities, and is mainly reflected in project promotion, policy implementation, technological development, and energy transformation costs. Farghali et al. proposed that there are differences in public perceptions and attitudes towards renewable energy, which provides a reference for analyzing its role in the construction of renewable energy systems [44]. Public acceptance is closely related to the cost of energy transition. The public’s active acceptance of changes in renewable energy and energy systems will promote the expansion of industrial scale, achieve economies of scale, and reduce costs. If the public has a high degree of acceptance of renewable energy, they will be more willing to support the construction of related projects, such as participating in the installation of distributed photovoltaic systems, providing a broader market space for the development of renewable energy, which is conducive to the realization of goals such as the installed capacity of renewable energy in the model. At the same time, public support will also prompt the government to more actively promote the transformation of the energy system, introduce more favorable policies, create a good policy environment for the implementation of the model, and a large number of renewable energy projects can be implemented, but in the process of project promotion, it still faces challenges brought by some social factors. Local residents may be reserved for the project due to concerns about changes in land use, changes in the ecological environment, the economic burden, and the complexity of transformation. This requires that social factors be fully considered during the project planning and implementation process, strengthen communication with the public, and take reasonable compensation and ecological protection measures to increase public acceptance. In addition, if the public has low acceptance of energy system changes, it may not cooperate with energy-saving measures, do not participate in demand response, etc., which will affect the overall operating efficiency of the energy system and make it difficult for the model to achieve the expected results.

Third, education will affect the acceptance of renewable energy technologies. Differentiation in technology cognition leads to differentiation of adoption behavior. A survey by Shandong University shows that the accuracy of the cognition of the “energy-saving principle” of heat pumps by farmers with high school or above is 2.1 times that of farmers with junior high school or below, which directly leads to the significantly higher use rate of heat pumps in Jiaodong than in southwestern Shandong, with a usage rate of 45% and 12% respectively. Education investment is related to long-term technical decision-making. Jiaodong high-income families pay more attention to education investment and increase technology acceptance through intergenerational knowledge transfer. For example, among the 2,000 families participating in photovoltaic training in Penglai District, Yantai, families with a college degree have a mastery rate of operation and maintenance knowledge after photovoltaic installation, and the fault handling efficiency is 60% higher than that of ordinary families. The reaction of renewable energy education on human capital and technical training promotes labor skills upgrading. The “photovoltaic technology training” of the Shandong Provincial Department of Agriculture and Rural Affairs covers 12,000 farmers in Shouguang, Weifang, of which 35% of the laborers with high school education and above have transformed into “rural energy technicians”, with an average monthly income of 2,000 yuan, driving the proportion of rural laborers with high school education and above in counties to increase from 28% to 35%. This cycle of “skill improvement-income growth-education investment” further strengthens the advantages of regional education. Knowledge spillover effect narrows the education gap. After the power grid renovation in the Yellow River Beach area, the promotion of electric heating technology was accompanied by “household training”, which increased the energy technology cognition scores of households with junior high school education and below 42% among the 500 families participating in the training in Leling City, Dezhou. Although it is still lower than the Jiaodong level, it narrowed the gap with the high-education area and laid the foundation for subsequent technology adoption.

Fourth: Overharvesting of wood biomass may lead to soil carbon loss. It is recommended to conduct ecological impact monitoring and sustainable raw material management, implement a biomass raw material sustainability certification system, limit biomass utilization from primary forests, and give priority to the use of agricultural waste and fast-growing energy crops. Establish a long-term ecological monitoring network to evaluate the impact of biomass development on soil, water quality, and biodiversity, and avoid ecological overdrafts.

Fifth, the energy acquisition model is related to the infrastructure of renewable energy deployment. The spatial differences in power grid infrastructure, the rural power grid coverage rate around Jinan is extremely high, providing stable support for photovoltaic grid connection, while the power grid coverage rate in the Yellow River Beach area of Liaocheng is only 92%, resulting in 20% of photovoltaic installed capacity being unable to be fully connected to the grid due to “voltage instability”, which restricts the enthusiasm for deployment. The wind power permeability in weak areas of the southwestern Shandong power grid is 40% lower than that in Jiaodong. The core reason is that the transmission line capacity is insufficient. The path dependence of traditional energy use inertia, the straw utilization rate of Heze farmers is 65%, and the coal dependence is high. Even after the power grid is renovated, 43% of households still reject photovoltaics because of “lower traditional energy costs”. The traditional energy utilization rate in rural Jiaodong is only 12%, the basis for power replacement is good, and the resistance to renewable energy deployment is smaller. Renewable energy has transformed and upgraded the energy acquisition model, and the power grid upgrade and energy structure transformation are coordinated. The construction of Dongying offshore wind farm has driven the transformation of power grids in surrounding villages, not only ensuring wind power grid connection, but also promoting the increase of the proportion of electric heating users from 18% to 41%, forming a virtuous cycle of “renewable energy power generation-grid optimization-clean energy consumption”, and the regional energy acquisition model has shifted from “traditional fuel-led” to “power-based”. Distributed energy reshapes the rural energy ecology. Through the “promotion of photovoltaics in the whole county”, Weifang Shouguang has transformed the rural household energy acquisition model from “single power supply in the power grid” to “spontaneous self-use + residual electricity access”. In 2023, the average electricity bill expenditure of farmers decreased by 35%. At the same time, through planting under photovoltaic panels (such as edible fungi), a “energy + agriculture” compound model was formed, and the land utilization rate increased by 200%, completely changing the separation state between traditional energy acquisition and agricultural production.

Applicability of model scope: Because the model parameters are mainly established in rural areas of Shandong Province, the model is mainly applicable to agricultural areas of the North China Plain (such as Shandong and Henan). Because the grass-grain ratio parameters are based on the calibration of northern staple grain crops (wheat, corn), the grass-grain ratio needs to be recalibrated for southern rice-producing areas. Priority is applicable to the coordinated evaluation of biomass and solar energy, and special modules need to be supplemented for other renewable energy such as wind energy and geothermal energy. This model is based on 2024 data. This model is evaluated based on prefecture-level cities and existing technologies. It has policy and technology limitations. Future research should be combined with actual conditions. Based on the above research, Table 7 briefly summarizes the future development paths of 16 prefectures and cities in Shandong Province according to the four major types.

**Table 7:** Summary of development paths of 16 cities in Shandong Province.

Classification Dimension	Double-high potential zone (Weifang, Jining, Binzhou, Heze, Dezhou)	Solar energy advantage zone (Yantai, Linyi, Dongying)	Biomass Advantage Zone (Liaocheng)	Resource-scarce areas (Jinan, Qingdao, Zibo, Zaozhuang, Tai’an, Weihai, Rizhao)
Resource Features	Agricultural waste and lighting resources are both abundant, with a total annual power generation of more than 200000 GWh.	It has superior lighting, limited biomass resources, and a total annual power generation of 107000 GWh.	Characteristic agriculture is developed, Rizhao is weak, and the annual total power generation is 31000 GWh	The urbanization rate is high, the natural endowment is insufficient, and it depends on external green electricity.
Core development path	Build a three-dimensional energy supply network of “agricultural and optical complementarity + straw power generation” and develop the “field energy + roof photovoltaic” model	Explore “Photo-hydrogen storage integration” and focus on photovoltaics and coastal wind power hydrogen production in saline-alkali lands.	Strengthen the cogeneration of biomass, and develop distributed energy based on characteristic agricultural waste.	Implement cross-regional green electricity allocation and building energy efficiency upgrades, and promote distributed photovoltaic and green electricity transactions.
Key implementation directions	Plant shade-tolerant energy crops under photovoltaic panels and build a distributed straw cogeneration center; Heze plans the “Thousand Villages Photovoltaic + Ten Thousand Acres of Energy Crops” demonstration zone.	Dongying’s saline-alkali land layout “Photovoltaic + energy storage + green hydrogen” project, hydrogen production is used for industrial replenishment. Yantai and Linyi promote “fishing and light complementary” centralized photovoltaic bases.	Build large-scale biomass gasification cogeneration projects to provide centralized heating for townships. Promote “biogas + photovoltaic” microgrid, and make organic fertilizer by manure slag.	Establish a cross-regional green electricity trading mechanism and purchase Shandong western green electricity. Forced to promote the integration of building photovoltaics, with the coverage rate of newly built rural houses ≥60%.
Technological innovation	Develop an intelligent linkage system for photovoltaic and biomass gasification furnaces, and dynamically adjust power supply priorities. Pilot the “Photovoltaic + Biomass” comprehensive energy station, and the comprehensive efficiency has been increased to 70%.	Pilot the “Photovoltaic direct supply hydrogen production” technology, with an efficiency target of more than 75%; A supporting flywheel energy storage system is used to smooth the fluctuations of photovoltaic.	Introduce high-temperature gasification coupled with carbon capture technology (CCUS) to achieve “negative carbon emissions” heating; Optimize the biogas fermentation process and increase gas production efficiency by 20%.	Develop a “virtual power plant” management platform to participate in peak shaving by aggregated and distributing energy; Promote “optical storage, direct and soft” buildings to intelligently adjust the electricity load.
Policy Tools	Establish a special subsidy for “multi-energy complementarity”,; Operation subsidies will be provided to straw storage stations.	Green hydrogen production enterprises are allowed to participate in spot power trading, and peak-to-valley price spreads are recovered; Land for photovoltaic projects enjoys agricultural land policy preferential treatment.	Implement “heat-based supplementation” and biomass heating subsidies; Tax exemption and tax reduction policies for specialty agricultural waste utilization projects.	Cross-regional green power transactions will eliminate transmission loss fees, reducing power purchase costs by 10%−15%; The subsidy for the photovoltaic renovation of rural houses is 200 yuan per square meter.
Core Challenge	Straw storage and storage are coordinated across departments (agriculture, energy, and environmental protection), with a long approval cycle; The initial investment of large-scale projects is high and the financing channels are single.	The corrosion resistance cost of photovoltaic modules in saline-alkali land increases by 15%−20%; The green hydrogen industry chain is insufficient and the terminal application scenarios are limited.	The collection radius of characteristic agricultural waste is large and the transportation cost is high; The heating demand for cogeneration projects fluctuates greatly in winter and the utilization rate of equipment is low.	The power grid infrastructure is weak, 20% of photovoltaic installed capacity cannot be fully connected to the grid due to unstable voltage; Traditional energy dependence is strong, and farmers are low in their willingness to transform.
Cracking strategies	Establish a provincial joint approval platform, and shorten the approval cycle to 3 months. Issuing “Biomass + Photovoltaic” green bonds to lower the financing threshold.	Adopt salt spray photovoltaic modules, with a life span of up to 25 years; The government takes the lead in establishing a “Green Hydrogen - Transportation” demonstration corridor to cultivate market demand.	Layout distributed storage and storage sites, with a radius controlled within 5 kilometers; Develop a multi-purpose system of “heating + agricultural product drying” to improve equipment utilization.	The rural power grid upgrade plan will be launched, and the average distribution capacity of households will reach 4.5kVA in 2026. The “scattered coal replacement” subsidy is implemented, and the equipment purchase price subsidy is 30%.
Implementation Roadmap (2025–2030)	2025-2026: Launch 5 “agricultural and optical complementarity + straw power generation” demonstration projects, with a total installed capacity of 500MW; 2027-2028: Build a multi-energy, complementary, integrated energy service platform; 2029-2030: Renewable energy accounts for 35%.	2025-2026: Dongying launches the first “photo hydrogen storage” pilot project, producing 5,000 tons of hydrogen per year; 2027-2028: Green hydrogen production capacity reaches 50,000 tons/year; 2029-2030: Hydrogen energy heavy truck applications account for more than 10%.	2025-2026: Construction of 2 large-scale biomass cogeneration projects, with heating coverage of 100,000 households; 2027-2028: The installed capacity of biogas power generation reaches 50MW; 2029-2030: Biomass heating accounts for more than 20%.	2025-2026: Complete the photovoltaic renovation of 500,000 rural houses and build 100 community energy storage stations; 2027-2028: Cross-regional green electricity transactions reach 20 billion kWh/year; 2029-2030: Renewable energy accounts for 25%.
Expected results	Form a circular ecosystem of “power generation - heating - agriculture”, with a 3-fold increase in energy output per unit of land and a 40% reduction in carbon emissions.	Build a “photovoltaic-hydrogen-industry” industrial chain, and the cost of green hydrogen is reduced to 15 yuan/kg, replacing 20% of industrial hydrogen.	Achieve closed-loop utilization of “agricultural waste - energy - organic fertilizer”, reduce carbon emissions of rural heating by 50%, and increase farmers’ annual income by 2,000 yuan.	The reliability of power supply in the power grid has increased to 99.8%, farmers’ electricity bills have decreased by 35%, and building energy consumption has decreased by 30%.

## 5. Conclusions

In order to realize the “double carbon target”, this study evaluates the rural renewable energy potential through the grass-to-grain ratio method and GIS technology in sixteen cities in Shandong Province, and finds that biomass and solar energy show significant regional differences: The biomass power generation potential of Heze, Dezhou and other major agricultural areas exceeds 16,000 GWh, the annual capacity of photovoltaic in the north-central part of the country, such as Dongying and Weifang, exceeds 35,000 GWh, while Weihai and Rizhao, which have a high rate of urbanization, need to rely on the external transfer of green power. Based on the four-quadrant model of resources, differentiated paths are proposed: Weifang and other five cities to build a multi-energy complementary “three-dimensional energy supply network”, Dongying and other three cities to develop “light storage and hydrogen integration” system, Liaocheng to dig deep into the potential of biomass cogeneration, and Jinan and other seven cities to promote the inter-regional green power deployment and building photovoltaic transformation. Jinan and other seven cities have made efforts to promote inter-regional green power deployment and building photovoltaic transformation.

There will be potential deviations in the model that should be noted. First, the model relies on data quality and coverage, emphasizing potential assessments and path frameworks rather than precise project-level predictions. As well as the cutting operations of ArcGIS software, they may cause some errors. Because of the limitations of the time difference between research and reality, the time intervals in statistical yearbook statistics have lags in technological progress, such as the application of the latest photovoltaic modules, which will potentially affect the accuracy of the model.

The GIS-four-quadrant model constructed in this study breaks through the static and singular limitations of traditional rural energy assessment. Through multi-energy collaborative quantification and space-time complementarity analysis, three aspects of innovation are achieved:

First of all, dynamic adaptability, based on GIS spatial data fusion and parameterized simulation, corrects resource potential and avoids development risks in slope >15% or ecologically sensitive areas;

Secondly, it is resilience-oriented to identify the climate adaptation potential of regional energy combinations through the abundance-complementarity matrix of biomass and photovoltaics;

The third is policy operability, and proposes the “four types of personalized paths” to provide customized transformation plans for areas with significant differences in resource endowment and urbanization rates.

Research suggests implementing multiple policies. First, the formulation of regionally appropriate energy development strategies. Secondly, technical training and infrastructure should be strengthened to enhance utilization efficiency. Thirdly, it is necessary to establish a cross-domain synergistic mechanism to optimize the allocation of resources, so as to build a multi-level renewable energy system and help rural revitalization and carbon neutrality goals to be achieved in a synergistic manner. Fourth, climate risk assessment tools need to be included, such as GIS superimposed on extreme weather probability layers, and climate risks must be shared through insurance mechanisms. Fifth, establish a rural energy database in order to achieve long-term policy guidance and data monitoring.

## Supporting information

S1 Data. (XLSX)

S2 Tabulation data. (XLSX)
